# The Effects of Cocoons of *Larinus Hedenborgi* (Coleoptera: Curculionidae) Extracts on Inflammation and Clinical Outcomes in Patients With Coronavirus Disease 2019: A Double-Blinded Randomized Controlled Clinical Trial

**DOI:** 10.1016/j.curtheres.2025.100811

**Published:** 2025-08-05

**Authors:** Mahdi Keshani, Sayid Mahdi Mirghazanfari, Naseh Pahlavani, Faezeh Baniyaghoobi, Vahid Hadi, Mohammad Bagherniya, Zahra Heidari, Amirhossein Sahebkar, Mohsen Mohajeri, Saeid Hadi

**Affiliations:** 1 Nutrition and Food Security Research Center and Department of Community Nutrition, School of Nutrition and Food Science, Isfahan University of Medical Sciences, Isfahan, Iran; 2 Departments of Physiology and Persian Medicine, Faculty of Medicine, AJA University of Medical Sciences, Tehran, Iran; 3 Health Sciences Research Center, Torbat Heydariyeh University of Medical Sciences, Torbat Heydariyeh, Iran; 4 Social Determinants of Health Research Center, Torbat Heydariyeh University of Medical Sciences, Torbat Heydariyeh, Iran; 5 Department of Military Nursing, Faculty of Nursing, AJA University of Medical Sciences, Tehran, Iran; 6 Department of Biochemistry and Nutrition, Faculty of Medicine, AJA University of Medical Sciences, Tehran, Iran; 7 Department of Biostatistics and Epidemiology, School of Health, Isfahan University of Medical Sciences, Isfahan, Iran; 8 Applied Biomedical Research Center, Mashhad University of Medical Sciences, Mashhad, Iran; 9 Biotechnology Research Center, Pharmaceutical Technology Institute, Mashhad University of Medical Sciences, Mashhad, Iran; 10 Department of Traditional Medicine, School of Persian Medicine, Iran University of Medical University, Tehran, Iran

**Keywords:** COVID-19, inflammation, *Larinus* spp., traditional medicine, *Trehala manna*

## Abstract

**Background:**

Severe acute respiratory syndrome coronavirus 2, the causative agent of coronavirus disease 2019 (COVID-19), is an inflammatory disease that manifests with symptoms including dry cough, fever, myalgia, and even pneumonia. Despite antiviral treatments, no definitive therapy has been proven effective. *Trehala manna* (TM), the edible cocoon of *Larinus hedenborgi* (Coleoptera: Curculionidae) weevil, as a natural product, is traditionally used to alleviate respiratory symptoms because of its antibacterial, anti-inflammatory, and antifungal properties.

**Objective:**

The current study aimed to determine the effects of TM add-on treatment on inflammatory biomarkers and some clinical outcomes in patients with COVID-19.

**Methods:**

The present study was a randomized controlled trial conducted on 60 patients who were randomly allocated to TM (5 g, twice a day) or a placebo for 1 week. The main outcomes of the current study include C-reactive protein (CRP) level, erythrocyte sedimentation rate (ESR), blood urea nitrogen level, creatinine level, fasting blood sugar level, systolic blood pressure, and visual analog scale (VAS) score for cough, which were evaluated at both the beginning and end of the study.

**Results:**

Our finding revealed that CRP level, ESR, blood urea nitrogen level, creatinine level, fasting blood sugar level, systolic blood pressure, and VAS score for cough were significantly reduced in the TM group after intervention (*P* < 0.05), and CRP level, ESR, VAS score for cough, and fever were significantly reduced in the TM group compared with the control after 7 days (*P* < 0.05).

**Conclusions:**

*Trehala mann* is a natural remedy with potential as an adjunctive therapy for reducing inflammatory biomarkers and improving certain clinical symptoms in patients with COVID-19. No adverse effects were observed during the trial. Iranian Registry of Clinical Trials identifier: IRCT20211029052904N1.

## Graphical abstract

**Figure:**
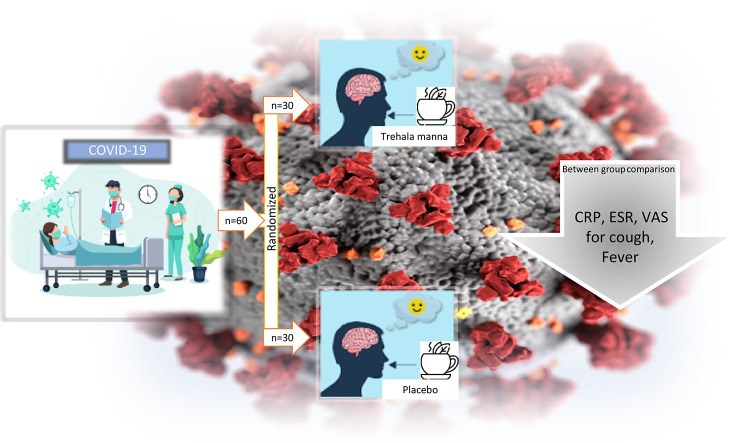

## Introduction

In late 2019, a novel virus emerged in Wuhan, China, and spread rapidly worldwide. Severe acute respiratory syndrome coronavirus 2 (SARS-CoV-2) is the causative virus for coronavirus disease 2019 (COVID-19).^1^ According to the World Health Organization data (January 9, 2022), approximately 304 million cases have been reported globally and approximately 5.4 million deaths have occurred, and many countries and territories have been involved.^2^ According to the latest report from this organization during the 4-week reporting period (September 16 to October 13, 2024), weekly SARS-CoV-2 polymerase chain reaction (PCR) positivity changed from 5.0% in the first week of the reporting period to 6.1% in the last week, with a weekly mean of 14,506 specimens tested across 90 countries.^3^

The virus spreads by aerosol droplets between people and enters pneumocytes in the respiratory system by binding to angiotensin-converting enzyme 2 receptors. Then, the virus is duplicated many times, and therefore, cells are damaged. After the last phase, damaged cells secrete cytokines and inflammation biomarkers into the bloodstream, called a cytokine storm in the innate immune response phase. Cytokine storms lead to overstimulation of the immune system, potentially progressing to acute respiratory distress syndrome, organ dysfunction, and death.^4^ Coronavirus disease 2019 is responsible for economic, social, and health difficulties. Respiratory, cardiovascular, mental, and neurological issues, such as anxiety, depression, tremors, or seizures, can result from COVID-19 infection.^5^^,^^6^ Coronavirus disease 2019 common symptoms include dry cough, dyspnea, fever, fatigue, myalgia, and even pneumonia or respiratory distress syndrome.^7^ Many health organizations and pharmacologic institutions are in an attempt to develop a new generation of drugs and vaccines to relieve or prevent the various symptoms of COVID-19.^8^

Traditional medicine, natural products, medicinal herbs, and their derivatives have been reported to have several beneficial effects on patients with COVID-19.^9^, ^10^, ^11^ The use of natural remedies to alleviate respiratory symptoms has a long history in traditional Iranian medicine (TIM).^12^ In the TIM, manna was used in different medicinal conditions for treatment or prevention, such as infant jaundice,^13^, ^14^, ^15^, ^16^ constipation, and as a laxative that accelerates the passage of meconium.^16^ There are several different types of mannas, which are *Astragalus manna* (Gesengebin), *Khansar manna, Herbaceous manna, Pajorak manna* (*Purgative manna* or Shir-e-khesht), *Camel’s thorn manna* (Taranjebin), *Willow manna* (Bidkhesht), Shekar-sorkh, and *Trehala manna* (TM).^17^^,^^18^ Some types of carbohydrates were found in manna, such as glucose, trehalose, and mannitol. Manna contains phenolic compounds, flavonoids, and micronutrients, which have different beneficial effects.^19^, ^20^, ^21^ In TIM, manna is the most frequent part of plants that dissolve in boiling water for use.^22^

*Trehala manna* has been highlighted in the Avicenna Pharmacopoeia, Al-Qanun, and Pharmacopoeia Persica of Fr. Angelus.^23^ *Trehala manna* (also known as *Echinops manna*, Insect manna, Shekar Tiqhal, or Shekar Tiqal) has an oval shape, 1.5 cm long and 1 cm wide, with a rough outer surface and a smooth inner surface.^18^^,^^24^ *Trehala manna* is a safe-to-eat and chalky-like cocoon of *Larinus hedenborgi* weevils (Coleoptera: Curculionidae) that emanated from the outgrowth of the larval nutrition secretion activities on the petioles of *Echinops cephalotes* DC. (EC) (globe thistles, Asteraceae) plants. Materials required for the cocoon construction are processed in the larval Malpighian tubules and are condensed in the hindgut before molting. In some areas in Asia (Iran, Iraq, Syria, Turkey, and Transcaucasia), the ripe cocoons, which have a sweet taste, are used up by the natives as food and a remedy. The cocoons and thistles are nomenclature as Shekar Tiqal (or Shekar Tighal) and Khar Shekar, respectively, in local parlance. *Trehala manna* is traditionally used to treat a variety of diseases, including fever and constipation, the common cold, sore throats, and influenza. Its actions are believed to be mediated through the enhancement of the human immune system and have antibacterial and antiviral activities.^23^^,^^25^, ^26^, ^27^ Studies have reported that TM has antibacterial, anti-inflammatory, and antifungal activities.^17^^,^^25^^,^^28^ *Trehala manna* is composed of cellulose, starch, a small amount of fat, and a large amount of trehalose (approximately 25%–30%) and is rich in magnesium. Biochemical components include compounds of saponins, steroids, cyanogenic glycosides, tannins, and alkaloids.^17^^,^^25^^,^^28^

Several studies have demonstrated the anti-inflammatory and antioxidant effects of TM, including its role in modulating cytokine storms and improving immune responses.^18^^,^^29^ However, its application in clinical settings, particularly in patients with COVID-19, has not been adequately explored. This study aimed to fill this gap by providing clinical evidence of TM’s efficacy.

We designed a parallel randomized, double-blind, placebo-controlled clinical trial to evaluate the effects of cocoons of *L hedenborgi* (Coleoptera: Curculionidae) extracts as adjunctive therapy on inflammation and clinical outcomes in patients with COVID-19.

## Materials and Methods

### Trial design

A superiority parallel, randomized, double-blind, controlled clinical trial based on the Consolidated Standards of Reporting Trials guidelines^30^ and according to the guidelines laid down in the Declaration of Helsinki was implemented in Imam Reza Hospital, Tehran, Iran, between May 2022 and October 2022. The study protocol has been approved by the Research Ethics Committees of AJA University of Medical Sciences (approval ID: IR.AJUMS.REC.1400.253) and was also registered on the Iranian Registry of Clinical Trials (unique ID: IRCT20211029052904N1; December 2021; available on https://fa.irct.ir/trial/59668). The research method was explained to the patients before enrollment, and informed consent was obtained.

### Participants

Eligibility criteria were as follows: patients aged 20 to 75 years with moderate COVID-19 according to the real-time PCR (RT-PCR) test who were admitted to the hospital COVID unit who were nourished orally, and who provided informed consent (by patients or their legal guardians) were included.

Chest radiograph at admission revealed typical radiological findings of COVID-19 pneumonia, with extensive bilateral ground-glass opacities; standard treatment was initiated before receiving the nasopharyngeal swab test results (RT-PCR, confirming SARS-CoV-2 infection). As mentioned, chest scans were taken at baseline for early recognition of infection and at least on another day of intervention for patient monitoring. The diagnosis of SARS-CoV-2 infection was confirmed based on participants’ RT-PCR tests, and then patients were recruited. Patients with moderate COVID-19 with a positive PCR test result, with symptomatic presentation (eg, fever, cough, and pulmonary involvement on chest radiograph), and without the need for oxygen supplementation were recruited.

Patients who were pregnant, breastfeeding, undergoing dialysis, or admitted to the intensive care unit (ICU) with severe and progressive COVID-19 or ICU admission, diffuse intravascular coagulation, or requiring frequent blood transfusions; patients with significant comorbidities such as chronic kidney disease, cardiovascular disease, cancer, or any other chronic diseases; and any other herbal medicine usage were excluded.

### Randomization, concealment mechanisms, allocation, and blinding

At first, the principal researcher (M.K.) clarified the potential beneficial effects of TM on the eligible patients and obtained written consent. Randomization was performed using a computer-generated sequence by an independent researcher who was not involved in the study. Allocation was concealed using sealed, opaque envelopes prepared by a clinical pharmacologist. The principal researcher was blinded to the group assignments throughout the trial. Standard protocol treatment was implemented for all patients; our interventions were added to these without altering the usual treatment strategy. *Trehala manna* and placebo have been stored by a blinded clinical pharmacologist in a package with the same shape and color and then labeled as 1 or 2. Therefore, participants, investigators, and data analysts were blinded to the study. Then, eligible participants were randomly assigned in a 1:1 ratio to receive either TM or a placebo. Balanced randomization was generated using an online software by a person who was not involved in the study. Investigators and participants were blinded to the treatment allocation.

Sealed and opaque envelopes prepared by the clinical pharmacologist determine package content. Packages that differed only in whether they were labeled 1 or 2 were prepared and provided to the principal investigator without specifying their contents. Another team researcher generated an allocation sequence of trial participants.

### Intervention and preparation of materials

*Trehala manna* decoction was prepared in the way that Persian traditional healers make crude TM decoction.^18^ *Trehala manna* was collected from a single geographic region (Fars province, Iran) and during a defined harvesting period (spring 2022). The material was authenticated by a botanist and stored under controlled conditions. Glucose, trehalose, and mannitol are the principal carbohydrate components of TM. Phenolic compounds, flavonoids, and micronutrients are also other components. *Trehala manna* is composed of cellulose, starch, a small amount of fat, and a large amount of trehalose (approximately 25%–30%) and is rich in magnesium. Biochemical components include compounds of saponins, steroids, cyanogenic glycosides, tannins, and alkaloids.^17^^,^^22^^,^^25^^,^^28^^,^^31^

*Trehala manna* had been dissolved in boiling distilled water, 5 g in 100 mL water, while being stirred for 30 minutes and then consumed versus a placebo. The placebo was prepared by a pharmacist to resemble TM in shape, odor, and taste. After receiving a written agreement and outlining the study conditions, all patients who met the inclusion criteria were invited to participate during the study period. *Trehala manna* or placebo was administered by mouth 2 times a day at 9:00 and 21:00 for 7 days (participants received 10 g TM or placebo per day in total). Return intervention packages were used for assessing adherence to the intervention. After the follow-up duration, if any participant consumed <80% of the packages (<11 packages), they were excluded from the trial. All patients received identical background treatment per national COVID-19 guidelines during the trial, including: antiviral therapy: remdesivir or favipiravir (as indicated). Supportive care includes hydration, antipyretics (eg, acetaminophen), and monitoring. No concurrent immunomodulators (eg, dexamethasone) were administered to avoid confounding. *Trehala manna*/placebo was added uniformly to this regimen.

### Sample size

The sample size of 60‍ participants (30 in the intervention group and 30 in the placebo group) was calculated based on the Pouladzadeh et al^32^ study with a power of 80%, a type I error of 0.05, an SD of 28 mg/L, and a standardized effect size of 0.7.

### Outcomes

Study outcome evaluators and laboratory staff were unaware of intervention allocation. Changes in C-reactive protein (CRP) level and erythrocyte sedimentation rate (ESR) were assessed as primary outcomes. Differences in blood urea nitrogen level, creatinine level, fasting blood sugar level, white blood cell (WBC) count, prothrombin time, partial thromboplastin time, systolic blood pressure, diastolic blood pressure, fever, and visual analogue scale (VAS) score for cough were assessed as ancillary outcomes. Participant characteristics include sex, weight, and height appraised at baseline.

Approximately 10 mL volume of fasting blood sample was drained before and after the study and then centrifuged, with the serum separated from the sediment and preserved at a temperature of −80°C. Study variables were measured with standard clinical chemistry techniques in the main 501-AJA (Imam Reza) hospital laboratory. Blood pressure obtained from vital signs monitoring device. Visual analogue scale score for cough and fever (using a standard thermometer through the mouth) was evaluated by M.K. on days 1 and 7.

### Statistical methods

Data were entered into SPSS software version 16 for analysis. The skewness test and Q-Q plot were applied to assess the normal distribution of variables. Quantitative and qualitative variables were reported as mean (SD) and number (percentage), respectively. Variable changes were reported as mean (SE). The descriptive statistic indices were used to analyze the data, such as percentage, confidence interval, frequency, and mean (SD), and also inferential statistical tests (Wilcoxon-Mann-Whitney, χ^2^, Fisher exact, Q-Q plot, ANOVA, and skewness). Baseline characteristics of the participants were compared between the groups, using an independent samples *t* test and Pearson’s χ^2^ test, where applicable. ANCOVA was used to detect any differences between the 2 groups at the end of the study and adjusted for baseline values and body mass index (BMI). The logarithmic transformation approach was applied to variables with an abnormal distribution. A *P* value of <0.05 was considered significant.

## Results

### Participant’s flow diagram and demographic data

A total of 190 inpatients were assessed for eligibility; 102 patients were excluded for not meeting the inclusion criteria, and 28 persons refused to participate in the study. Finally, 60 patients were randomly assigned to the intervention (10 g of TM daily) or control (10 g of placebo daily) group. It is important to note that we had not dropped out of the intervention or control groups. Therefore, 30 participants in the TM group and 30 participants in the placebo group completed the trial (all participants consumed >80% of the interventions). The Consolidated Standards of Reporting Trials diagram of the current study is presented in the Figure. The baseline characteristics of participants are detailed in Table 1. The mean (SD) age of the patients was 51.10 (15.49) years. Thirty-five of the participants were male, and 25 of them were female. As shown in Table 1, at baseline, there were no significant differences in demographic variables between the 2 groups (*P* > 0.05) except for weight and BMI (*P* < 0.05). Although TM is expected to have tolerability,^25^ a physician monitored participants daily for possible adverse effects related to TM or the placebo. No adverse effects were reported or observed in any participants, and all patients completed the intervention without interruption.

**Fig:**
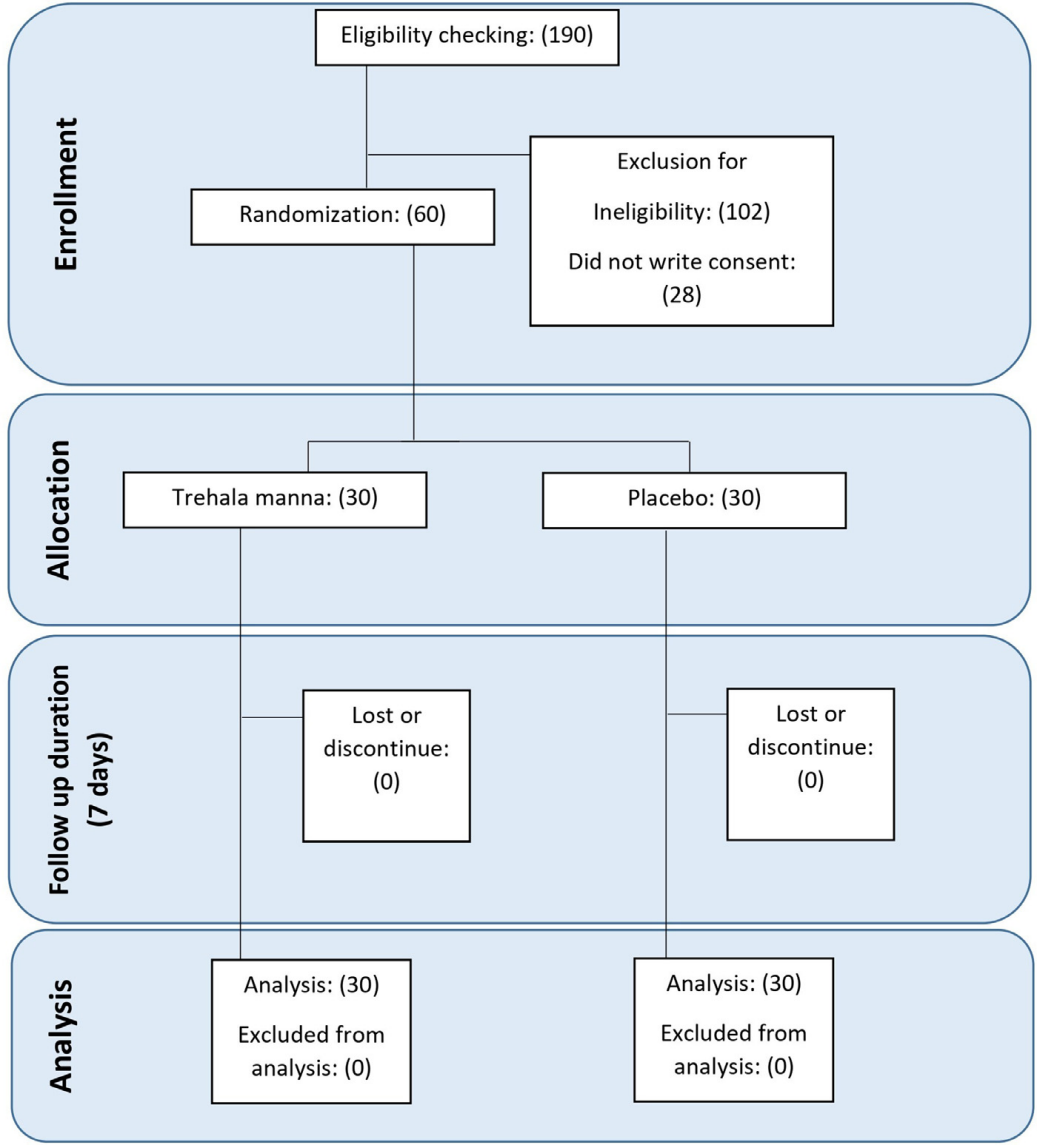
Consolidated Standards of Reporting Trials study flow diagram.

**Table 1:** Comparison of baseline characteristics between the TM and control groups.

Variable*	TM group (n = 30)	Control group (n = 30)	*P* value†
Age (y)	50.87 (13.97)	51.33 (17.12)	0.908
Sex)			
Male	16 (53.3%)	19 (63.3%)	0.432
Female	14 (46.7%)	11 (36.7%)	
Wight (kg)	74.17 (10.99)	65.30 (18.71)	0.030
Height (cm)	166.50 (8.84)	165.07 (12.98)	0.619
BMI (kg/m^2^)	26.77 (3.54)	23.54 (4.50)	0.003

BMI = body mass index; TM = *Trehala manna*.

⁎ Values are mean (SD), except for categorical variables, which are presented as n (%).

† Resulted from independent samples *t* test for continuous and Pearson χ^2^ test for categorical variables.

### The effects of TM on primary outcomes

Compared with the baseline, CRP level (*P* < 0.001) and ESR (*P* < 0.001) were significantly reduced in the intervention group after 7 days. The between-group analysis found that TM significantly reduced CRP level in the intervention group compared with the control group (*P* = 0.004, adjusted for baseline value), but between-group differences for ESR did not reach the significant level (*P* = 0.127, adjusted for baseline value) (Table 2).

**Table 2:** Inflammatory variables in the TM and control groups at baseline and the end of the trial.

Variable*	Timeline	TM (n = 30)	Placebo (n = 30)	*P* value†	Adjusted *P* value‡	Adjusted *P* value^§^
CRP (mg/dL)	Baseline	43.03 (14.01)	38.69 (14.86)	0.226	0.004	0.009
	7th day	13.45 (14.67)	18.83 (14.81)	0.021		
	Changes	−29.58 (2.99)	−19.86 (3.50)			
	*P* value║	<0.001	<0.001			
ESR (mg/dL)	Baseline	35.30 (13.92)	35.73 (11.13)	0.373	0.127	0.077
	7th day	23.57 (9.42)	27.10 (12.02)	0.210		
	Changes	−11.73 (1.64)	−8.63 (1.94)			
	*P* value	<0.001	<0.001			

CRP = C-reactive protein; ESR = erythrocyte sedimentation rate; TM = *Trehala manna*.

⁎ Values are mean (SD) except for mean changes (SE).

† Obtained from independent sample *t* test.

‡ Obtained from analysis of covariance in the adjusted models (adjusted for baseline value). ^§^Obtained from analysis of covariance in the adjusted models (adjusted for baseline value and body mass index).

║ Obtained from paired sample *t* test.

When further adjustment was conducted for BMI, the between-group differences for CRP were again statistically significant (*P* = 0.009). This analysis found that ESR was reduced at a marginally significant level (*P* = 0.077) (Table 2).

### The effects of TM on secondary outcomes

Blood urea nitrogen level (*P* < 0.001), creatinine level (*P* < 0.001), fasting blood sugar level (*P* < 0.001), fever (*P* < 0.001), systolic blood pressure (*P* = 0.016), and VAS score for cough (*P* < 0.001) were reduced statistically significantly, and WBC count (*P* < 0.001), hemoglobin level (*P* = 0.002), prothrombin time (*P* < 0.001), and partial thromboplastin time (*P* = 0.014) were significantly increased in the TM group after intervention. Between-group differences revealed that VAS score for cough was reduced in the TM group compared with the control group (*P* = 0.001). Further adjustment for BMI did not change the significance. The mean levels of clinical and laboratory variables are shown in Table 3.

**Table 3:** Monitoring laboratory variables in the TM and control groups at baseline and the end of the trial.

Variable*	Timeline	TM (n = 30)	Placebo (n = 30)	*P* value†	Adjusted *P* value‡	Adjusted *P* value§
BUN level (mg/dL)	Baseline	43.90 (13.50)	40.00 (16.44)	0.166	0.879	0.685
	7th day	30.90 (14.54)	28.90 (10.36)	0.746		
	Changes	−13.00 (2.32)	−11.10 (3.35)			
	*P* value║	<0.001	0.003			
Cr level (mg/dL)	Baseline	1.98 (0.65)	2.00 (0.80)	0.869	0.449	0.448
	7th day	1.65 (0.58)	1.53 (0.31)	0.509		
	Changes	−0.33 (0.07)	−0.47 (0.12)			
	*P* value║	<0.001	0.001			
FBS level (mg/dL)	Baseline	156.10 (69.74)	154.23 (52.62)	0.841	0.554	0.342
	7th day	137.13 (55.50)	132.27 (41.00)	0.930		
	Changes	−18.97 (4.34)	−21.97 (5.62)			
	*P* value║	<0.001	0.001			
WBC count (× 10^9^/L)	Baseline	5.20 (0.88)	5.09 (0.95)	0.334	0.372	0.594
	7th day	6.48 (1.64)	5.92 (1.29)	0.317		
	Change*s*	1.28 (0.23)	0.83 (0.23)			
	*P* value║	<0.001	0.001			
Hb level (g/dL)	Baselin*e*	13.38 (1.86)	14.18 (2.27)	0.109	0.321	0.357
	7th day	13.60 (1.78)	14.15 (2.17)	0.228		
	Changes	0.22 (0.06)	−0.03 (0.17)			
	*P* value║	0.002	0.858			
Hct (%)	Baseline	36.89 (4.16)	37.66 (4.59)	0.396	0.980	0.961
	7th day	37.00 (3.86)	37.62 (4.56)	0.572		
	Changes	0.11 (0.40)	−0.04 (0.46)			
	*P* value║	0.781	0.926			
MCV (fl)	Baseline	86.66 (3.08)	87.42 (5.64)	0.492	0.493	0.689
	7th day	86.99 (2.96)	87.56 (5.69)	0.632		
	Changes	0.33 (0.17)	0.14 (0.10)			
	*P* value║	0.058	0.475		0.735	0.967
MCH (pg)	Baseline	30.52 (2.86)	31.49 (3.02)	0.203		
	7th day	30.95 (2.55)	31.51 (3.14)	0.457		
	Changes	0.44 (0.45)	0.01 (0.27)			
	*P* valu*e*║	0.337	0.965			
MCHC (g/dL)	Baseline	34.04 (2.83)	35.36 (1.40)	0.027	0.594	0.551
	7th day	34.62 (2.36)	35.00 (1.43)	0.463		
	Changes	0.58 (0.46)	−0.37 (0.26)			
	*P* value║	0.216	0.165			
Plt (/mL)	Baseline	180.33 (80.33)	175.80 (49.67)	0.318	0.977	0.751
	7th day	184.47 (82.13)	180.40 (65.73)	0.833		
	Changes	4.13 (4.65)	4.60 (8.60)			
	*P* value║	0.381	0.597			
PT (s)	Baseline	12.56 (0.79)	13.06 (1.25)	0.074	0.362	0.264
	7th day	13.08 (0.52)	13.14 (1.56)	0.834		
	Changes	0.51 (0.12)	0.08 (0.25)			
	*P* value║	<0.001	0.746			
PTT (s)	Baseline	26.83 (2.74)	28.37 (3.93)	0.085	0.888	0.878
	7th day	28.12 (2.73)	29.37 (4.86)	0.227		
	Changes	1.29 (0.49)	1.00 (0.66)			
	*P* value║	0.014	0.139			
Na (mg/dL)	Baseline	132.93 (3.03)	133.27 (3.35)	0.111	0.665	0.948
	7th day	134.83 (3.08)	135.17 (4.22)	0.728		
	Changes	1.90 (0.40)	0.90 (0.84)			
	*P* value║	<0.001	0.291			
Ca (mg/dL)	Baseline	8.74 (0.50)	8.71 (0.47)	0.812	<0.001	<0.001
	7th day	8.88 (0.46)	8.57 (0.42)	0.009		
	Changes	0.13 (0.05)	−0.14 (0.07)			
	*P* value║	0.020	0.041			
Total bilirubin level (mg/dL)	Baseline	0.92 (0.23)	0.84 (0.32)	0.267	0.128	0.155
	7th day	0.93 (0.28)	0.80 (0.30)	0.069		
	Changes	0.02 (0.19)	−0.04 (0.03)			
	*P* value║	0.606	0.236			
Direct bilirubin level (mg/dL)	Baseline	0.33 (0.13)	0.32 (0.16)	0.865	0.235	0.085
	7th day	0.35 (0.03)	0.32 (0.16)	0.454		
	Change*s*	0.02 (0.02)	0.00 (0.01)			
	*P* value║	0.237	0.749			
Fever (°C)	Baseline	38.34 (0.83)	38.02 (0.63)	0.102	0.038	0.177
	7th day	36.98 (0.36)	37.10 (0.37)	0.213		
	Changes	−1.35 (0.15)	−0.92 (0.09)			
	*P* value║	<0.001	<0.001			
SBP (mmHg)	Baseline	137.53 (21.20)	142.00 (15.29)	0.731	0.855	0.991
	7th day	129.30 (13.28)	131.50 (11.53)	0.496		
	Changes	−8.23 (3.23)	−10.50 (2.29)			
	*P* valu*e*║	0.016	<0.001			
DBP (mmHg)	Baseline	88.37 (10.27)	92.23 (11.29)	0.130	0.862	0.771
	7th day	85.50 (8.07)	87.17 (9.80)	0.475		
	Changes	−2.87 (1.78)	−5.07 (1.40)			
	*P* value║	0.119	0.001			
RR	Baseline	19.53 (2.79)	20.60 (3.55)	0.201	0.191	0.223
	7th day	19.10 (1.65)	20.30 (3.21)	0.075		
	Changes	−0.43 (0.49)	−0.30 (0.57)			
	*P* value║	0.382	0.601			
HR	Baseline	89.93 (17.40)	90.47 (12.40)	0.948	0.081	0.078
	7th day	83.40 (20.92)	90.37 (11.81)	0.163		
	Changes	−6.53 (3.80)	−0.10 (1.60)			
	*P* value║	0.084	0.912			
O_2_ saturation (%)	Baseline	92.60 (2.43)	92.43 (2.34)	0.091	0.380	0.269
	7th day	96.13 (2.43)	95.63 (2.14)	0.361		
	Changes	3.53 (0.40)	3.20 (0.47)			
	*P* value║	<0.001	<0.001			
VAS score for cough	Baseline	4.90 (1.45)	5.07 (1.60)	0.673	0.001	<0.001
	7th day	1.90 (1.30)	2.87 (1.38)	0.007		
	Changes	−3.00 (0.17)	−2.20 (0.22)			
	*P* value║	<0.001	<0.001			

BUN = blood urea nitrogen; Ca = calcium; Cr *=* creatinine; DBP *=* diastolic blood pressure; FBS *=* fasting blood sugar; Hb = hemoglobin; Hct = hematocrit; HR *=* heart rate; MCH = mean corpuscular hemoglobin; MCHC = mean corpuscular hemoglobin concentration; MCV = mean corpuscular volume; Na = sodium; Plt = platelet; PT *=* prothrombin time; PTT *=* partial thromboplastin time; RR *=* respiratory rate; SBP *=* systolic blood pressure; TM *= Trehala manna*; VAS *=* visual analogue scale; WBC *=* white blood cell.

⁎ Values are mean (SD) except for mean changes (SE).

† Obtained from independent sample *t* test.

‡ Obtained from analysis of covariance in the adjusted models (adjusted for baseline value).

§ Obtained from analysis of covariance in the adjusted models (adjusted for baseline value and body mass index).

║ Obtained from paired sample *t* test.

## Discussion

To the best of our knowledge, no clinical trials have evaluated the effectiveness of extracts from cocoons of *L hedenborgi* (Coleoptera: Curculionidae) in patients with COVID-19, making this study the first of its kind. According to the results of the current study, TM can reduce CRP level, ESR, fever, and VAS score for cough in the TM group compared with the placebo group.

The significant reduction in CRP level and ESR observed in this study aligns with previous research demonstrating the anti-inflammatory effects of TM.^25^^,^^29^ Furthermore, the improvement in clinical symptoms such as cough and fever is consistent with findings from traditional medicine interventions that target cytokine storms in viral infections.^33^

Inflammation is a vital and complex process that our immune system uses to defend itself; however, heightened inflammation may lead to disease exacerbation. Many nonsteroidal anti-inflammatory drugs act against inflammation by inhibiting the metabolism of arachidonic acid, but they can exhibit some secondary negative effects.^34^ According to the World Health Organization, approximately 3-quartile people throughout the world use traditional herbal medical plants to relieve their ailments. Medical plants, for their efficacy, inexpensive costs, low side effects, and high availability, have received a lot of attention.^34^

Despite limited knowledge about COVID-19 pathophysiology, it is associated with increased oxidative stress and inflammation.^35^ Besides, cough, fever, and sore throat are the main symptoms.^28^^,^^36^

A study by Hajibeygi et al^37^ that was conducted on non-ICU patients with COVID-19 demonstrated that a mixture of TIM can reduce CRP levels meaningfully in 5 days.^37^ Another randomized clinical trial by Xiong et al,^33^ which was carried out on 42 inpatients with COVID-19, found that Xuanfei Baidu Decoction for 7 days can meaningfully reduce CRP level and ESR as well as fever, cough, fatigue, and loss of appetite and can increase WBC count.^33^

An animal study by Heidari et al^25^ revealed that TM had promising effects on metabolic profile and had a protective effect on the liver of diabetic rats. Another study reported that phytochemical extracts of TM have antioxidant effects.^17^ Additionally, TM can regulate liver and intestinal function and acts as a laxative, expectorant, antiaging agent, and antioxidant agent more powerful than vitamins C and E. In cellular models, its extract neutralized membrane lipid oxidation of red blood cells and inhibited the production of reactive oxygen species and proinflammatory cytokines (interleukin 6 and interleukin 8).^31^^,^^38^

A study by Hamedi et al^18^ isolated TM macromolecules and revealed that it has immunomodulatory effects. The water-soluble and water-insoluble fractions of several carbohydrates of TM have different biological effects, such as strong cytotoxic and proliferative activity. Additionally, TM immunoregulatory properties on splenocytes and macrophages were also declared.^18^^,^^29^. Moreover, an in vitro study by Ahmadabad et al^29^ revealed that TM extract can modify the ratio of type 1–to–type 2 T helper cells, which regulates the immune system.^29^.

Trehalose constitutes approximately one-third of the weight of TM,^25^ and recent evidence revealed that trehalose has positive effects on the spectrum of diseases, such as infectious and metabolic diseases.^39^, ^40^, ^41^, ^42^ Trehalose is a nonreducing disaccharide that is made up of 2 units of glucose and is known as an autophagy enhancer. Currently, trehalose is known for its antiviral effects. It can induce interferons, facilitate lysosomal degradation of the intracellular virus, decrease viral entrance to cells by reducing the expression of host cell surface proteins, which the viruses attach to, decrease cathepsin activity, and reduce inflammation.^43^ Trehalose acts as an antioxidant and neuroprotective agent. It can inhibit infectious diseases (acquired immunodeficiency syndrome, tuberculosis, and cytomegalovirus infection) by inducing endolysosomal degradation pathways and autophagy.^44^ Additionally, trehalose is marked as extremely low toxicity and well-tolerated. Trehalose acts as an anti-inflammatory agent and can inhibit harmful oxidative stress partially owing to the increment of endogenous antioxidant defense represented by the Nrf2 protein.^45^ Bodies of evidence revealed that trehalose may have beneficial prophylactic effects like hydroxychloroquine against SARS-CoV-2 by modulating the factors related to virus entry.^43^ Trehalose can induce type 1 interferons, facilitate lysosomal degradation of the intracellular virus, and decrease cathepsin activity, so it is recognized as an anti-inflammatory and antiviral agent.^43^ It can potentially be used in the remission of COVID-19 infection and some other viral infections. Antiviral effects of trehalose on other viral infections, such as human cytomegalovirus and varicella-zoster virus, have been indicated in vitro.^46^

In addition to trehalose, there are other important ingredients, such as alkaloids, saponins, polyphenols, carotenoids, phytosterols, and tannins in TM.^17^^,^^47^ Polyphenols and flavonoids have shown antiviral effects and inhibitory effects on ACE2, human cytomegalovirus, and the DNA synthesis of viruses.^48^ Luteolin, one of the well-known polyphenols, attaches to the surface protein of coronavirus and can block the entrance of the virus into the cells. Naringenin, another polyphenol found in many plants, exhibited very strong antiviral activity. It binds to the spike glycoprotein of the coronavirus more powerfully than remdesivir.^49^

### Strengths

Previous studies on TM have primarily focused on its antioxidant and anti-inflammatory properties in vitro or in animal models, leaving a gap in clinical evidence.^18^ This study, being a double-blind randomized controlled trial, overcomes these limitations by providing robust clinical data on the efficacy and safety of TM as an adjunctive therapy in patients with COVID-19.

### Limitations

Despite the novelty of the current trial, some limitations should be considered (due to the budgetary restriction), including the small number of patients and the lack of measuring other inflammatory biomarkers and cytokines. Another limitation of the current study was that only mild–to-moderate COVID-19 cases were enrolled, which limits the generalizability of the results to severe cases. All participants in this trial were unvaccinated against COVID-19, as the study was conducted before the widespread availability of vaccines in our region. This was confirmed via self-reporting and medical records at enrollment. Thus, vaccination status could not confound the observed effects of the extract on inflammatory or clinical outcomes. Data on prior vaccinations for non-COVID-19 viral pathogens were not collected. Although randomization likely balanced these factors between groups, future studies should document such variables to explore potential interactions with immune outcomes. Additionally, we did not document detailed gynecologic histories, including menopausal status or number of children, for women participating in the study. Regarding future perspectives, we suggest that further randomized clinical trials with more participants be performed, especially with the control of hormonal status.

## Conclusion

*Trehala manna*, when used as an adjunct to standard care, was related to a significant reduction in inflammatory biomarkers (CRP level and ESR) and some clinical symptoms (fever and cough) in patients with mild-to-moderate COVID-19. In addition, no adverse effects were reported. Further studies with larger sample sizes are needed to confirm these findings.

## Declaration of competing interest

The authors declare that they have no competing financial interests or personal relationships that might have influenced the work in this paper.
